# Biogeochemistry of upland to wetland soils, sediments, and surface waters across Mid-Atlantic and Great Lakes coastal interfaces

**DOI:** 10.1038/s41597-023-02548-7

**Published:** 2023-11-24

**Authors:** Allison N. Myers-Pigg, Stephanie C. Pennington, Khadijah K. Homolka, Allison M. Lewis, Opal Otenburg, Kaizad F. Patel, Peter Regier, Madison Bowe, Maxim I. Boyanov, Nathan A. Conroy, Donnie J. Day, Cooper G. Norris, Edward J. O’Loughlin, Jesse Alan Roebuck, Lucie Stetten, Vanessa L. Bailey, Kenneth M. Kemner, Nicholas D. Ward, EXCHANGE Consortium, Silver Alford, Michael P. Back, Andy Baldwin, Jade Bolinger, Jacob A. Cianci-Gaskill, Matthew J. Cooper, Alex Demeo, Kyle Derby, Derek Detweiler, Suzanne Devres-Zimmerman, Erin Eberhard, Keryn Gedan, LeeAnn Haaf, Erin Johnson, Aliya Khan, Matthew L. Kirwan, Payton Kittaka, Erika Koontz, Adam Langley, Riley Leff, Scott Lerberg, Sairah Y. Malkin, Amy M. Marcarelli, Steven E. McMurray, Tyler Messerschmidt, Taylor C. Michael, Holly A. Michael, Elizabeth C. Minor, Brian Moye, Thomas J. Mozdzer, Scott Neubauer, Andrea Pain, Michael Philben, Evan Phillips, Dannielle Pratt, Lauren Sage, Daniel Sandborn, Stacy Smith, Alexander Smith, Samina Soin-Voshell, Bongkeun Song, Amanda Sprague-Getsy, Kari St. Laurent, Lorie Staver, Alice Stearns, Rebecca Swerida, Ethan J. Theuerkauf, Katherine Tully, Rodrigo Vargas, Elizabeth Watson, Coreen Weilminster

**Affiliations:** 1 Pacific Northwest National Laboratory, Marine and Coastal Research Laboratory, Sequim, Washington USA; 2 The University of Toledo, Department of Environmental Sciences, Toledo, Ohio USA; 3 Pacific Northwest National Laboratory, Joint Global Change Research Institute, College Park, Maryland USA; 4 Lawrence Berkeley National Laboratory, Environmental Genomics, and Systems Biology, Berkeley, California USA; 5 Pacific Northwest National Laboratory, Richland, Washington USA; 6 Argonne National Laboratory, Lemont, Illinois USA; 7 Institute of Chemical Engineering, Bulgarian Academy of Sciences, Sophia, Bulgaria; 8 Los Alamos National Laboratory, Los Alamos, New Mexico USA; 9 The University of Washington, Department of Oceanography, Seattle, Washington USA; 33 Present Address: Port Gamble S’Klallam Tribe, Kingston, WA USA; 10 University of Maryland, College Park, MD USA; 11 Kent State University, Kent, OH USA; 12 Old Woman Creek National Estuarine Research Reserve, Huron, OH USA; 13 Central Michigan University, Institute for Great Lakes Research, Mount Pleasant, MI USA; 14 Virginia Institute of Marine Science, Gloucester Point, VA USA; 15 Maryland Department of Natural Resources, Annapolis, MD USA; 16 Hope College, Holland, MI USA; 17 Michigan Technological University, Houghton, MI USA; 18 George Washington University, Washington, DC USA; 19 Partnership for the Delaware Estuary, Wilmington, DE USA; 20 Villanova University, Villanova, PA USA; 21 University of Minnesota, St. Paul, MN USA; 22 Smithsonian Environmental Research Center, Edgewater, MD USA; 23 University of Maryland Center for Environmental Science, Cambridge, MD USA; 24 University of Delaware, Newark, DE USA; 25 Large Lakes Observatory, University of Minnesota, Duluth, MN USA; 26 Bryn Mawr College, Bryn Mawr, PA USA; 27 Virginia Commonwealth University, Richmond, VA USA; 28 Delaware State University, Dover, DE USA; 29 Chesapeake Bay National Estuarine Research Reserve - Maryland, Annapolis, MD USA; 30 Delaware Department of Natural Resources and Environmental Control, Dover, DE USA; 31 Michigan State University, East Lansing, MI USA; 32 Stony Brook University, Stony Brook, NY USA

**Keywords:** Biogeochemistry, Environmental sciences

## Abstract

Transferable and mechanistic understanding of cross-scale interactions is necessary to predict how coastal systems respond to global change. Cohesive datasets across geographically distributed sites can be used to examine how transferable a mechanistic understanding of coastal ecosystem control points is. To address the above research objectives, data were collected by the EXploration of Coastal Hydrobiogeochemistry Across a Network of Gradients and Experiments (EXCHANGE) Consortium – a regionally distributed network of researchers that collaborated on experimental design, methodology, collection, analysis, and publication. The EXCHANGE Consortium collected samples from 52 coastal terrestrial-aquatic interfaces (TAIs) during Fall of 2021. At each TAI, samples collected include soils from across a transverse elevation gradient (i.e., coastal upland forest, transitional forest, and wetland soils), surface waters, and nearshore sediments across research sites in the Great Lakes and Mid-Atlantic regions (Chesapeake and Delaware Bays) of the continental USA. The first campaign measures surface water quality parameters, bulk geochemical parameters on water, soil, and sediment samples, and physicochemical parameters of sediment and soil.

## Background & Summary

The structure and function of coastal ecosystems vary considerably across relatively small spatial scales, resulting in dynamic hydrological and biogeochemical behaviors along the gradient of coastal upland, wetland, and surface water environments^1,2^. Insight into drivers of spatial heterogeneity can be elucidated by linking biogeochemical data with ecosystem properties^3,4^, enabling scientific discovery and model parameterization, such as furthering mechanistic understanding of coastal ecosystems and improving uncertainty constraints of coastal models^1,5^.

Open access and interoperable coastal biogeochemical datasets are needed to predict how coastal systems will respond to global change^3,6^. The Great Lakes and Mid-Atlantic regions have a wealth of long-term monitoring programs hosting open access datasets, such as the National Estuarine Research Reserve^7^, the Great Lakes Wetland Monitoring Program^8^, and the Chesapeake Bay Program^9^, among others. However, the synthesis of existing data streams across traditional ecosystem and disciplinary boundaries is still relatively sparse^1,5^. Here, we describe datasets collected as part of EXCHANGE Campaign 1 (EC1), which establishes a baseline understanding of the chemical forms and distribution of carbon and nutrients across coastal terrestrial-aquatic interface (TAI) research sites in the Great Lakes and Mid-Atlantic regions (Chesapeake and Delaware Bays) of the continental USA that can be utilized to conduct synthesis. EXCHANGE adds to existing efforts in these regions by developing a consortium of regional researchers interested in exchanging knowledge and information, with a molecular level focus that spans upland to aquatic domains, that can contribute to understanding of how coastal systems will respond to global change.

**Fig. 1:**
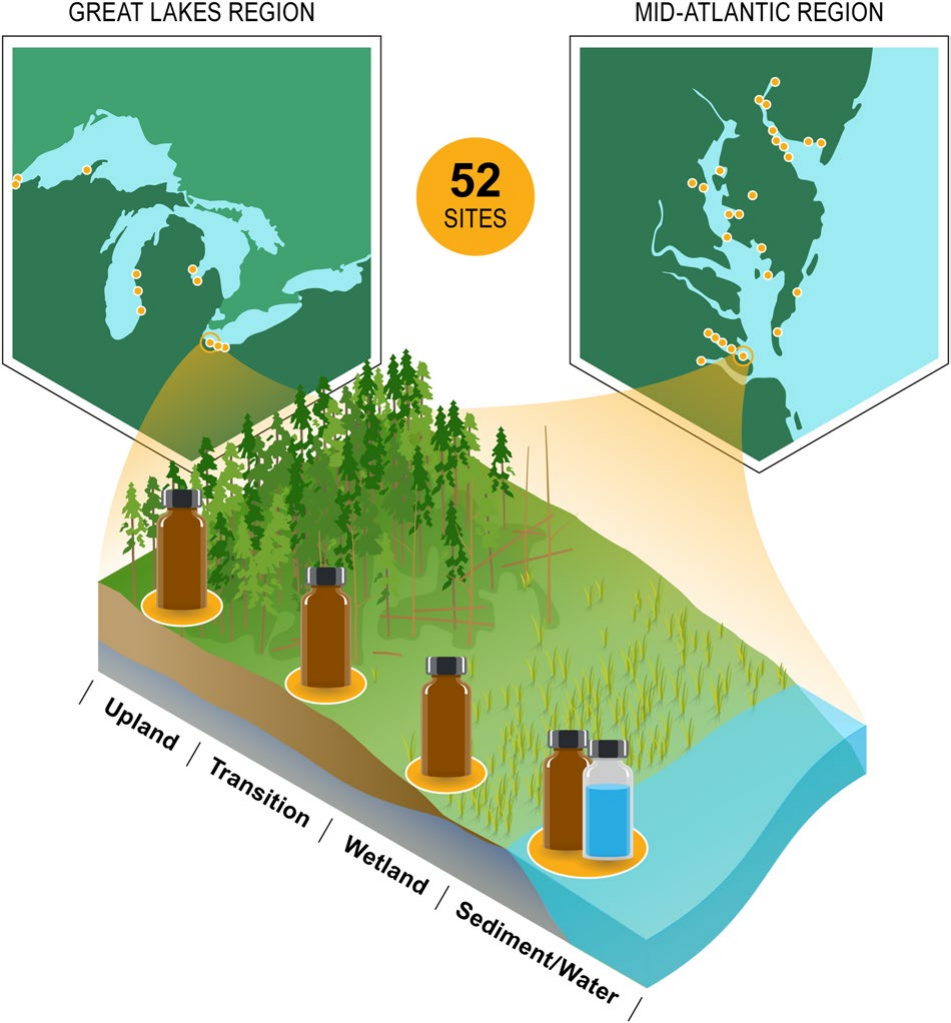
EXCHANGE Campaign 1 sites were located in the Great Lakes and Mid-Atlantic Regions. 52 terrestrial-aquatic interfaces were sampled, from uplands to nearby waters (lake, estuary, stream, river, etc) for surface soils, sediments, and water samples.

In the Fall of 2021, the EXCHANGE Consortium collected samples from 52 coastal terrestrial-aquatic interfaces (TAIs). At each of these TAI sites, the consortium collected soil samples from across a transverse elevation gradient, which included soils from coastal upland forests, transitional forests, and wetlands. The consortium also collected surface water and nearshore sediment samples adjacent to the transverse elevation gradient (Fig. 1). Samples collected from EC1 were analyzed for bulk geochemical parameters, bulk physicochemical parameters, organic matter characteristics, and redox-sensitive elements. These datasets are useful in evaluating the physicochemical factors that drive spatial variations in the cycling of organic matter across coastal terrestrial-aquatic interfaces (TAIs). They also facilitate an understanding of the biogeochemical control points in coastal ecosystems that can be assessed for their transferability across different coastal systems. Here, we describe version one (v1) of the key baseline datasets that are currently published open access^10^. We also describe additional datasets that will be published in subsequent versioning of the data package in the Supplementary Information.

## Methods

### Sampling and processing

#### Sampling design

The experimental design of EC1 was developed via workshops (following open science principles^11^) from conception to data analysis and publication. Coastal researchers gathered virtually to design a spatially distributed sampling campaign across Great Lakes and Mid-Atlantic regions (Fig. 1). The EC1 consortium collected surface waters, soils, sediments, and site level metadata using standardized sampling kits. Following sample collection, all sample kits were shipped to the Marine and Coastal Research Laboratory (Sequim, WA), part of Pacific Northwest National Laboratory.

#### Site metadata

At each site, the EXCHANGE consortium collected standardized site metadata, such as latitude, longitude, and type of water system (e.g., estuary, lake). Additional site metadata, such as elevation and soil type, were extracted from publicly available databases (e.g., GoogleEarth) using site coordinates.

#### Surface waters

Field-filtered water – using 0.22 µm Sterivex syringe filters – was collected in vials for dissolved organic carbon (DOC), total dissolved nitrogen (TDN), common dissolved ions, stable water isotopes, and several organic matter characterization methods. Samples were filtered into vials in the field and preserved by freezing or storing at 4 °C until analyzed, depending on the analyte (Table 1). A 125 mL amber HDPE bottle of unfiltered water was collected with no headspace for pH, oxygen-reduction potential (ORP), alkalinity, and conductivity measurements of the surface waters. Unfiltered surface water samples were also collected in 1 L acid-cleaned HDPE amber bottles for total suspended solids and filtered to 0.2 µm in the lab, within 48 hours of collection. Lab-filtered 1 L grab samples were extracted for several organic matter characterization methods (e.g., high-resolution mass spectrometry) using standard solid phase extraction (SPE) procedures^12^. The filtered samples were stored at 4 °C until SPEs were completed, within 2 weeks of sample collection. Briefly, SPE was performed by passing one liter of sample through a 6 mL/1 g PPL SPE cartridge (Agilent PPL), after being acidified 24 hours before extraction to a pH of 2. Samples were then eluted in LC-MS grade methanol and were stored at −20 °C until analysis. Additional analysis beyond those reported herein (e.g. common dissolved ions and stable water isotopes) will be performed on archived filtered waters or SPE extracts as appropriate for the analysis method, and appended to future versions of the data package^10^.

**Table 1:** Collection methods, storage protocols, laboratory processing, and analytes by sample type.

Sample type	Collection	Storage Method	Additional preparation for storage or analysis	Analyses performed
Soil & Sediment	Intact cores (soils only)	4 °C	—	Bulk density, water retention curves, particle size analysis
	Grab samples	−20 °C	Lyophilize, sieve	Total carbon, total nitrogen, soil pH and conductivity, poorly crystalline iron
		4 °C	Sub-sample in anoxic environment	X-ray absorption spectroscopy
			—	Gravimetric water content
Water	Unfiltered grab samples	4 °C	—	Water quality (pH, ORP, alkalinity, conductivity)
			Filter in lab with GFF and then 0.2µm PES, filtrate run through solid phase extraction, extract stored at −80 °C	High-resolution mass spectrometry
			GFF filter dried at 45 °C, then stored at room temperature in desiccator	Total suspended solids
	Filtered grab samples	−20 °C	—	Common dissolved anions and cations
		4 °C	—	Dissolved organic carbon, total dissolved nitrogen, colored dissolved organic matter absorbance and fluorescence, water isotopes

#### Soils

Surface soils (top 5 cm of soil profile) were collected from the three transect locations (upland, transition, and wetland) from each TAI site. Soils were collected as intact cores (using HYPROP sampling rings, 5 cm diameter × 5 cm depth) and as surface grab samples (using 2.5 oz plastic (clear polypropylene) jars and plastic bags). The intact cores were refrigerated at 4 °C upon arrival to the laboratory. Subsamples of soil grabs samples were either immediately processed, frozen (−20 °C), or refrigerated, based on the analyses planned (Table 1). Frozen grab samples were freeze-dried, catalogued, and sieved to 5.6 mm before additional analyses (Supplementary Information; Figure S1; Table S1). Water retention curves, particle size analysis, X-ray absorption spectroscopy measurements, and any other additional analysis that will be performed will be appended to future versions of the data package (methods outlined in Supplementary Information)^10^.

**Table 2:** List of analytes located in each .zip folder of the current version (v1) of the data package.

Data Types	
*ReadMe*	
*Metadata*	Metadata taken at the time of sample collection (Collection Level Metadata)
	Metadata taken for each kit (Kit Level Metadata)
	Data taken during each sample collection (Collection Level)
	Sample catalog
	Data dictionary of each column present in data package (DD)
	File-level metadata of each file present in data package (FLMD)
	IGSN sample metadata (IGSN Metadata)
*Water Data*	water quality (pH, ORP, alkalinity, conductivity)
	total suspended solids (TSS)
	dissolved organic carbon (DOC)
	total dissolved nitrogen (TDN)
	high-resolution mass spectrometry (FT-ICR-MS)
	colored dissolved organic matter absorbance and fluorescence (CDOM)
*Sediment Data*	gravimetric water content (GWC)
*Soil Data*	gravimetric water content (GWC)
	bulk density (BD)
	soil pH and conductivity (pH, Cond)
	total carbon (TC)
	total nitrogen (TN)

#### Sediments

Surface sediments (i.e., top 5–10 cm of sediment) were collected into clear 2.5 oz polypropylene jars and frozen at −20 °C upon arrival for archival purposes. One full plastic bag of sediment was also collected for gravimetric water content (GWC) and was stored at 4 °C until analysis. Immediately upon arrival, subsamples from the sealed plastic bags were collected in minimal oxygen conditions and frozen for Fe X-ray absorption fine structure (XAFS) analysis. X-ray absorption spectroscopy measurements and analyses performed on sediment samples will be appended to future versions of the data package (methods outlined in Supplementary Information)^10^.

### Surface water analyses

#### Common water quality measurements (pH, ORP, conductivity, alkalinity)

Common water quality measurements (i.e., pH, ORP, conductivity, alkalinity) were performed on unfiltered surface water samples, within 24 hours of receiving. Samples were measured simultaneously for temperature, specific conductivity, oxidation-reduction potential, and alkalinity using a Mettler Toledo T7 auto-titrator equipped with an auto-sampler. Prior to starting each run and after every five samples, conductivity and pH sensors were checked with standards, and were recalibrated if outside the acceptable tolerance (+/− 1% for conductivity, and +/− 0.05 for pH). Conductivity was calibrated with a 50,000 μS/cm (+/− 1%) solution to cover the salinity range represented by samples (0 to ~35 PSU). pH was calibrated using a three-point calibration curve (using calibration solutions of pH 4.01, 7.00, and 10.00). Alkalinity was determined by titration with 0.02 N HCl to an endpoint of pH 4.00, following standard United States Geological Survey (USGS) procedures^13^. All water quality variables underwent quality control to flag values outside of sensor analytical ranges.

#### Dissolved organic carbon and total dissolved nitrogen

Field-filtered samples were stored at 4 °C until analyzed for dissolved organic carbon (DOC) and total dissolved nitrogen (TDN). DOC and TDN analyses were performed simultaneously, within one week of sample collection on a Total Organic Carbon Analyzer (Shimadzu TOC-L). DOC was measured as the best 3 of 5 injections, after in-line acidification with 1:12 hydrochloric acid, as non-purgeable organic carbon (NPOC) via catalytic combustion. TDN was measured by chemiluminescence as the best 3 of 5 injections. A combined carbon and nitrogen check standard was run every 10 samples; all values were within 10 ± 7% of the concentration for DOC and 6.5 ± 7% of the concentration for TDN. Peaks were disregarded if the coefficient of variation between replicate injections was greater than 2.0%. Data underwent quality control, including visual inspection of calibration curves, check standards, and sample peak shapes; values were flagged when they were outside of the calibration curve and instrument detection limit ranges.

#### Total suspended solids

Total suspended solids were measured on 1 L grab samples and filtered within 24 hours of sample collection, following Environmental Protection Agency (EPA) method 160.2^14^ with slight modifications. Samples were filtered through pre-combusted and pre-weighed glass fiber filters (GFF, nominal pore size of 0.7 µm). The filtrate was then filtered through 0.2 µm PES filters and stored at 4 °C until solid phase extraction (SPE) procedures were performed.

GF filters were dried in a 45 °C oven for 24–72 hours for TSS. Filters were dried until the filter mass was stable and stored in a desiccator for 24–48 hours after drying, until final weights were taken. Process blanks were filtered concurrently with sample filtering, and average blank signal was below detection. TSS were calculated gravimetrically as follows:

$$TSS\;mg/L=\frac{(Oven\;dry\;weight\;of\;sample\;and\;filter,in\;mg-oven\;dry\;weight\;of\;filter,in\;mg)\times 1000\;mL/L}{volume\;filtered\;in\;mL}$$

The volume filtered in mL was determined via mass and corrected for density of variable salinity waters using temperature, pressure and salinity data obtained from the titrator dataset with the package *gsw*^15^ in R version 4.2.1. When the common water quality measurements samples were not collected at a site, data were gap filled by taking the average of all adjacent kits. Data underwent further quality control to flag values below the blank and above the reported method detection limit for the EPA method^14^.

#### Colored dissolved organic matter absorbance and fluorescence

UV absorbance scans and excitation-emission matrices (EEMs) were collected simultaneously with an Aqualog (Horiba Scientific) on filtered sub-samples, which were stored at 4 °C until analysis. Absorbance was measured from 230 to 800 nm in 3 nm intervals, and blank corrected prior to exporting the data. EEMs were collected with the same wavelength constraints and further processed with drEEM toolbox v. 6.0 for Matlab^16^ (https://www.openfluor.org). EEMs processing included blank correction, inner filter correction^17^, and normalization to Raman Scatter units based on daily water Raman scans collected at an excitation of 350 nm.

#### High-resolution mass spectrometry

Aliquots of SPE extracts described in the sampling and processing methods for surface waters were normalized to a DOC concentration of 50 mg C/L prior to FTICR-MS analysis^18^. Spectra were collected at the Environmental Molecular Sciences Laboratory in Richland, WA, using a 12 Tesla (12 T) Bruker SolariX Fourier transform ion cyclotron resonance mass spectrometer (FTICR-MS) (Bruker, SolariX, Billerica, MA) with a custom direct infusion system (that performed two offline blanks between each sample) and an electrospray ionization (ESI) source. Data were acquired in negative mode with the needle voltage set to +4.0 kV and were collected from 150 m/z – 1000 m/z at 8 M. Three hundred scans were co-added for each sample and internally calibrated using OM homologous series separated by 14 Da (–CH_2_ groups). Mass measurement accuracy was generally within 1 ppm for singly charged ions across a broad m/z range (150 m/z - 1100 m/z). Raw spectra were converted to a list of m/z values using Bruker Data Analysis (version 5.0) by applying FTMS peak picker module with a signal-to-noise ratio (S/N) threshold set to 7 and absolute intensity threshold to the default value of 100. Chemical formulae were then assigned using Formularity^19^, an in-house software, following the Compound Identification Algorithm^20–22^. Criteria to assign chemical formulae included a S/N >7, and mass measurement error <0.5 ppm, taking into consideration the presence of C, H, O, N, S and P and excluding other elements^19^. Further processing of the data was done using the *fticrrr* R package^23^, including: (a) removing peaks <200 and >800 m/z, (b) removing peaks associated with ^13^C, and (c) blank correcting all spectra.

### Soil and sediment analyses

#### Gravimetric water content

Gravimetric water content (GWC) was determined, calculated and reported as the dry moisture content^24^. Field moist soil (~5 g) was dried in the oven at 100 °C for 24 hours. Weight loss was then calculated using the following equation:

$$gwc\left({\rm{ \% }}\right)=\frac{field\;moist\;weight-oven\;dry\;weight}{oven\;dry\;weight}\times 100$$

Dry weight basis is utilized herein to better indicate whether or not the soils were saturated^24^ across the broad spatial heterogeneity captured in EC1.

#### Bulk density

Bulk density was determined on intact cores collected in HYPROP rings, and calculated as:

$$bulk\;density(\,g/c{m}^{3})=\frac{dry\;weight}{soil\;volume}$$

Samples in the HYPROP rings were collected and maintained at field moisture, so the following conversion was applied to calculate the dry weight in the above equation:

$$dry\;weight=\frac{wet\;weight}{\left(GWC/100\right)+1}$$

#### Total carbon and nitrogen

Total carbon and nitrogen on a percent weight basis was determined via combustion and chromatographic separation using an ECS 8020 CHNS-O Elemental Analyzer (Orbit Technologies Pvt. Ltd.) equipped with a zero-blank electronic autosampler and thermal conductivity detector. Approximately 15 mg of freeze-dried, sieved, and homogenized soil were weighed into tin capsules. Reaction and reduction columns were packed according to operation manual specifications for C/N mode. For sample analysis, furnace temperatures were set to 980 °C for the reaction column, 650 °C for the reduction column, and 65 °C for the gas chromatograph. Carrier gas flow was held constant at ~110 ml/min. Standard reference sediments (SRM 1944; NY/NJ Waterway sediments) were run prior to each sample set, immediately following the calibration curve. We confirmed software peak detection, peak identification and integrations prior to exporting data. Calibration curves and final sample weight percentages were calculated in R Version 4.2.1 with the package *EnvStats*^25^.

#### Soil pH and conductivity

Soil pH and specific conductance were measured on freeze-dried and homogenized soils. Soil subsamples were shaken with deionized MilliQ water (1:10 weight:volume ratio) for 30 minutes and then analyzed using a Myron L 6PIIFCE pH and conductivity meter.

## Data Records

Data (for complete list, see Table 2) are permanently deposited on the open access repository Environmental Systems Science Data Infrastructure for a Virtual Ecosystem (ESS-DIVE)^26,27^, accessible at 10.15485/1960313^10^. Additional data types will be added to the ESS-DIVE data package as they are completed and will be version-controlled in the Change History section of *README.pdf*.

The structure of the data package is as follows:

Data Package Structure*

- ec1_metadata_v1.zip
  - *ec1_dd.csv*: a file-level data descriptor file containing a list of every column present in the data files
  - *ec1_flmd.csv*: a file-level data descriptor file containing a list of every file name present in the data package
  - *ec1_sample_catalog.csv*: a file containing a list of all samples and their collection status or information about methodological inconsistencies
  - *ec1_metadata_kitlevel.csv*
  - *ec1_metadata_collectionlevel.csv*
  - *ec1_data_collectionlevel.csv*
  - *ec1_igsn_metadata.csv*

- ec1_soil_v1.zip
- ec1_sediment_v1.zip
- ec1_water_v1.zip
- ec1_processingscripts_v1.zip

*Please note the ESS-DIVE data package will include additional versions as we add new data types and the version number on the data package will reflect the latest version.

*CSV file structure*

- [Campaign]_[Sample Type]_[Analyte]_[QC level].csv
  - Ex. *ec1_soil_tctn_L2.csv*
  - Ex. *ec1_metadata_kitlevel_L2.csv*
- All .csv dataset files contain the following first three identifying columns:
  - *campaign*: coordinated sampling effort, Ex. EC1
  - *kit_id*: unique identifier for each collection of samples from a given site, Ex. K001
  - *transect_location*: position along the coastal TAI transect (Fig. 1), Ex. wetland
*DAT file structure*
- [Kit_ID]_[Processing Step A]_[Processing Step…Z].dat
  - Ex. *K004_DilCorr_IFE_RamNorm.dat*
  - Ex. *K013_DilCorr_Abs.dat*
- All .dat dataset files are organized by Kit_ID and in matrices.

*Processing script structure*

- [Sample Type]_[Analyte].R
  - Ex. *soil_tctn.R*
  - Ex. *water_cdom.R*
- All processing scripts follow a standardized structure outlined in *template.R*

## Technical Validation

**Fig. 2:**
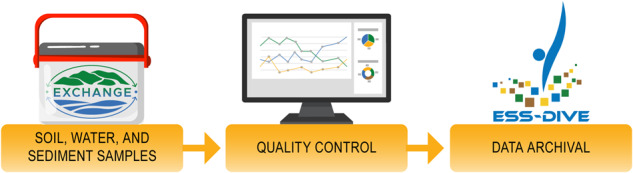
Workflow of quality control procedures. Samples are received from the consortium, then processed at the Marine and Coastal Research Laboratory (PNNL–Sequim, WA) for analyses, which then were shared with the consortium and the public on ESS-DIVE.

Technical validation steps were completed throughout the analysis process for each analyte (Fig. 2). Quality assurance of sample integrity was maintained from sample kit receiving, assuring that the quality of each sample was not compromised, by monitoring temperature and container quality upon kit arrival and stored properly for each analyte (Table 1). Instruments used to acquire EXCHANGE datasets were calibrated before each run and maintained using standard procedures for each instrument. Datasets were quality controlled following processing level designations (Table 3), inspired by the Ameriflux and Fluxnet programs^28,29^. For Level 1 (L1) datasets, flags are provided but are not applied. L1 datasets were screened for a secondary review and calculating the limit of detection ranges. Normal procedures for data quality were implemented, such as blank correction, etc, as appropriate. Analytical replicates are averaged, and outliers are also removed for L1 datasets. These datasets are archived on a Google Drive repository for additional data provenance and are accessible by the entire EXCHANGE consortium. For Level 2 (L2) datasets, all flags are applied to the L1 datasets, flagged data points removed, and data are summarized based on categorical variables (e.g., Transect Location, Kit ID). Datasets available on ESS-DIVE include L2 data for concentration-based datasets^10^.

**Table 3:** Data quality control levels and descriptions.

Level	Description
1	Flags are provided but not applied and LOD ranges. Normal QA/QC procedures implemented (e.g. blank correction, etc).
	Analytical averaging and outlier removal.
2	All flags applied to data.
	Analytical averaging and outlier removal.
	Data is summarized based on categorical variables (e.g. Transect Location, Kit ID)

We adopted the use of ESS-DIVE’s sample ID, file-level metadata, and CSV reporting formats^30–33^ to increase the usability of this data package and generate findable, accessible, interoperable and reusable (FAIR) data for the coastal science community^33^.

## Usage Notes

This dataset follows Creative Commons Attribution 4.0 licensing, making all data freely available to use and distribute via the ESS-DIVE repository. Additional analyses are being performed on these sample sets, methods detailing these analyses can be found in the Supplementary Information. The data package^10^ will be updated periodically with such additional datasets, found at the same DOI, with version numbers of the data package indicating new datasets are available.

EXCHANGE is an open science, community-driven program. We encourage those that use this data for subsequent analyses to deposit their code in an open source repository, which aids in furthering our collective knowledge about coastal interfaces.

### Supplementary information

Supplementary Information

## Data Availability

All code necessary to reproduce our Level 2 datasets are written in the open source R Statistical Software^34^ version 4.2.2 and are publicly available in our ESS-DIVE repository accessible at 10.15485/1960313^10^ in *ec1_processingscripts_v1.zip*. All scripts follow a standardized structure outlined in *template.R* and are named in the following format: [Sample Type]_[Analyte].R that correspond to their respective dataset name.
