# Supplementary material for: Biogeochemistry of upland to wetland soils, sediments, and surface waters across Mid-Atlantic and Great Lakes coastal interfaces
Source: Sci Data. 2023 Nov 24;10:822. doi: 10.1038/s41597-023-02548-7 (PMC10673855; doi:10.1038/s41597-023-02548-7)
Supplement: Supplementary file 1 — Supplementary Information [file 41597_2023_2548_MOESM1_ESM.pdf]

Supplementary Information for: Biogeochemistry of upland to wetland soils, sediments, and surface waters across coastal interfaces

Allison N. Myers-Pigg[1,2*], Stephanie C. Pennington[3*], Khadijah K. Homolka[1,4], Allison M. Lewis[5], Opal Otenburg[1], Kaizad F. Patel[6], Peter Regier[1], Madison Bowe[1], Maxim I. Boyanov[7,8], Nathan Conroy[9], Donnie J. Day[2], Cooper G. Norris[6], Edward J. O'Loughlin[7], Jesse Alan Roebuck Jr.[1], Lucie Stetten[7], Vanessa Bailey[6], Kenneth M. Kemner[7], Nicholas D. Ward[1,10], EXCHANGE Consortium[§]

Affiliations

[1] Pacific Northwest National Laboratory, Marine and Coastal Research Laboratory, Sequim, Washington, USA; [2] The University of Toledo, Department of Environmental Sciences, Toledo, Ohio, USA; [3] Pacific Northwest National Laboratory, Joint Global Change Research Institute, College Park, Maryland, USA; [4] Present address: Port Gamble S'Klallam Tribe, Kingston, WA, USA; [5] Lawrence Berkeley National Laboratory, Environmental Genomics, and Systems Biology, Berkeley, California, USA; [6] Pacific Northwest National Laboratory, Richland, Washington, USA; [7] Argonne National Laboratory, Lemont, Illinois, USA; [8] Institute of Chemical Engineering, Bulgarian Academy of Sciences, Sophia, Bulgaria; [9] Los Alamos National Laboratory, Los Alamos, New Mexico, USA; [10] The University of Washington, Department of Oceanography, Seattle, Washington, USA; * These authors contributed equally to this work; [§] A full list of members appears in the Author Contributions in the main text.

Corresponding Author(s):
Allison Myers-Pigg (allison.myers-pigg@pnnl.gov)
Stephanie Pennington (stephanie.pennington@pnnl.gov)

Table of Contents

Visual Soil and Sediment Characteristics……2
Additional Analyses……2
Water: Water Isotopes……3
Water: Common Dissolved Anions and Cations……3
Soil: Water Retention Curves……3
Soil: Particle Size Analysis……3
Soil: Poorly Crystalline Fe……4
Soil/Sediment: X-ray Absorption Spectroscopy (XAS) Measurements and Analyses……4
References……5
Supplementary Figures……6
Figure S1……6
Supplementary Tables……7
Table S1……7

Visual Soil and Sediment Characteristics

A visual classification system was developed which catalogs the heterogeneous characteristics of collected soils and sediments to inform sample selection for future analyses. Categories include type of plant litter type, soil/sediment color, pebble shape, root mass, root thickness, visibly identifiable minerals, visible organisms, shell condition, presence of visual iron oxidation, tactile estimation of relative cohesion, presence of plastics and visual estimation of sand content. Characteristics within a category were not mutually exclusive, with exception to the binary categories. Lyophilized samples were cataloged by the same individual during the dry sieving process to reduce subjectivity and maintain consistent interpretation (Figure S1).

Samples were cataloged as containing characteristics of the plant litter type or visible organisms categories if one or more characteristics were visually identified. Each sample was color matched to the colors defined in the 'Soil/Sediment Color' in Table S1 and was recorded as having an additional color if the sample contained streaks that differed from that of the matrix. Pebble shape was determined from lithified material that was 4-64 mm in size. Root mass and sand content were visually estimated based on distribution or approximate volume throughout the sample. Root thickness was defined as fine roots being <2 mm in thickness and coarse roots >2 mm in thickness. Visible minerals were constrained to mica and quartz because of their easily identifiable properties. Mica appeared as thin mineral flakes displaying a pearly luster on a cleavage face while quartz appeared as transparent grains displaying a vitreous luster and indistinct cleavage. Shell condition categorized any shell material as intact and/or broken; whole molluscs were recorded as intact. Relative soil and sediment cohesion was tested by breaking a large piece of sample between one's fingers and determined to be either 'easy to break apart' or 'hard to break apart'; samples were also classified as being 'aggregated' if soil aggregates were present. Samples that had little to no cohesion between particles were recorded as 'unconsolidated sand' or 'unconsolidated organic matter' depending on their composition. The binary categories of presence of visible iron oxidation and presence of microplastics were recorded as 'yes' if one or more instances of the characteristic were observed. Presence of visible iron oxidation was defined as any presence of a rust color in the samples.

Additional Analyses

Additional analyses to be released in the subsequent versions of the data package[1] include: a) total carbon (TC), total nitrogen (TN) on sediments, b) surface water isotopes, c) soil water retention curves, d) soil particle size analysis, e) soil poorly crystalline iron, f) X-ray Absorption Spectroscopy (XAS) measurements and analyses on soils and sediments, and g) surface water common dissolved cations and anions. Methodology for each of these data types can be found below. Data will be added to the data package when available[1]. Depending on sample volume remaining, additional analysis will be performed and added to future versions of the data package. This includes extractable nutrients, mineralogy (x-ray diffraction, XRD), and specific organic matter compound class identification (such as lignin phenols). These methodologies will be described in detail in subsequent works and added to the existing data package when available.

Water: Water Isotopes

Water isotope sub-samples were stored well sealed and at 4°C until analysis. Samples were analyzed for $\delta^{18}O$ and $\delta^{2}H$ on a Los Gatos Research (LGR) Liquid Water Isotope Analyzer. We used LGR Working Standards 1-5 to generate the calibration curve, and USGS 47 Reference Standard as the check standard to confirm quality of samples. We manually confirmed the quality of calibration curves, check standards, and standard deviations between sample injections prior to exporting data. The results were normalized to Vienna Standard Mean Ocean Water (VSMOW) before publication.

Water: Common Dissolved Anions and Cations

Field-filtered water samples were frozen at -20°C until analysis, then analyzed for seven common anions (F^-, Cl^-, Br^-, SO_4^{2-}, PO_4^{3-}, NO_2^-, $NO_3^{-)}$ and 6 common cations (K^+, Na^+, Mg^{2+}, Ca^{2+}, Li^+, NH_4^+) present in natural waters within one year of collection using ion chromatography on ThermoFisher Dionex ICS-6000 HPIC DP system. A thermal conductivity detector was used for all ions except nitrate and nitrite, which were detected using a UV detector [11]. Given the wide range in ion concentrations across analytes and within individual samples, ion samples were serially diluted to ensure each sample ion concentration was within the calibration curve range for that ion. Chromeleon software automatically identified peaks using manually defined retention times, based on commercially available standards. We visually confirmed software detection, peak identification, integrations, and the quality of calibration curves prior to exporting data. Ion data was dilution corrected and underwent quality control to flag values outside of the calibration curve and detection limit ranges.

Soil: Water Retention Curves

Intact cores were saturated and run on HYPROP devices to determine the water retention properties. Water retention curves were fitted using the Van Genuchten model on the measured data. For a subset of samples, we also measured water potential on the dry end of the curve using the WP4C dewpoint potentiameter, and these data were incorporated into the water retention curves.

Soil: Particle Size Analysis

Following water retention curve analysis, the oven-dry soils were analyzed for soil texture using the hydrometer method. Samples were subsampled (40 g) and sieved through 4-mm mesh and then pretreated with 30% hydrogen peroxide to remove organic matter. The pre-treated soil was mixed with 50 mL of 50 g/L sodium hexametaphosphate (HMP) and then the volume was made up to 1 L. Hydrometer measurements were taken at 90 minutes and 24 hours to calculate the percent clay. The sand fraction was determined by passing the soil slurry through 0.53 µm sieve. The % sand-silt-clay was used to determine the soil texture class.

Soil: Poorly Crystalline Fe

Poorly crystalline iron was extracted from soils using 0.5 M HCl and measured on a plate reader using a modified ferrozine method from Huang & Hall [2]. One gram of freeze-dried soil was shaken with 0.5 M HCl for 1 hour at 200 rpm at room temperature. The samples were then centrifuged at 7000 rcf for 15 minutes, and the supernatant was pipetted out and refrigerated until analysis. 10 µL of the extract was pipetted into 96-well plates, to which we added 10 µL of 0.5 M HCl, 20 µL of 10 % ascorbic acid (reducing agent, to convert Fe-III to Fe-II), and 100 µL of ferrozine color reagent prepared in HEPES buffer and adjusted to pH 8. Color was allowed to develop for 30 minutes and the absorbance was measured at 562 nm. Standards for Fe-II were developed using ferrous ammonium sulfate and diluting with 0.5 M HCl. By this technique, we calculated total extractable Fe in our samples (Fe-II + Fe-III).

Soil/Sediment: X-ray Absorption Spectroscopy (XAS) Measurements and Analyses

X-ray absorption spectroscopy at the Fe K-edge was used to determine Fe speciation (i.e., oxidation state and molecular environment) in soil and sediment samples from selected EC1 sites. X-ray absorption spectroscopy measurements were carried out at the MR-CAT/EnviroCAT bending-magnet beamline (Sector 10, Advanced Photon Source, USA), equipped with a Si(111) double crystal monochromator [3]. Fe K-edge x-ray absorption spectra were recorded in fluorescence mode using a 4-element Si drift detector (Hitachi Vortex ME-4) on selected soil and sediment samples. Energy calibration was established by setting the inflection point in the transmission spectrum of a metallic Fe foil to 7,112 eV and maintained afterwards by collecting data from the foil approximately every two hours. Measurements were performed at ambient temperature in a cell under constant flow of N_2, on unaltered (i.e., hydrated) soil/sediment samples mounted in 1.5 mm thick Teflon holders enclosed in Kapton film. All samples were mounted in an anoxic glove box (Coy Laboratory Products, Grass Lake, Michigan, 3–5% H_2 in N_2 and Pd catalyst to maintain O_2 in the box <1 ppm). For each sample, 5 to 10 XAS spectra were recorded. No differences were observed between the spectra taken from three locations on the same sample, indicating that no beam damage and no oxidation/reduction of the Fe-bearing phases occurred during the measurement.

A large set of Fe-references was used to interpret the EXAFS data, including spectra from mackinawite Fe(II)S, pyrite $Fe(II)S_2$, vivianite ($Fe(II)_3(PO_4)_2 \cdot 8H_2O$), siderite $Fe(II)CO_3$, carbonate green rust [$Fe(II)_4Fe(III)_2(OH)_{12}$].[$4H_2OxCO_3$], magnetite $Fe(II)Fe(III)_2O_4$, goethite α-Fe(III)O(OH), ferrihydrite $Fe(III)_{10}O_{14}(OH)_2$, hematite $Fe(III)_2O_3$, lepidocrocite γ-Fe(III)O(OH), Fe(II)-citrate, Fe(III)-citrate, Fe(III)-acetate, and native phyllosilicates harboring various Fe(II)/Fe(III) ratios : illite (IMt-2), nontronite (NG-1 and NAu-1), montmorillonite (SWy-2) and chlorite (CCa-2) [4–7]. These latter phyllosilicate minerals were also reacted with citrate-bicarbonate-dithionite (CBD) to reduce a fraction of their structural Fe^{3+} (Red- IMt-2, Red-NG-1, Red-NAu-1, Red-SWy-2, Red-CCa-2), according to the protocol described in Ilgen et al [8]. The polycrystalline Fe powders were mounted on the adhesive side of Kapton tape, and their absorption spectra were measured in transmission mode. The clay minerals were measured as

wet pastes mounted in the Teflon sample holders, in transmission and/or fluorescence mode depending on the Fe content and thickness of the sample.

For each sample, individual XAS spectra were merged, normalized and background subtracted using the Athena software [9], to obtain x-ray absorption near edge structure (XANES) and extended x-ray absorption fine structure (EXAFS) spectra. Principal Component Analyses (PCA) and Target Transformations (TT) were performed using the SIXPACK software [10] to evaluate the suitability of the Fe-references to describe the experimental k^3-weighted EXAFS spectra. Linear Combination-Least Square Fitting (LC-LSF) analyses were performed using the Athena software for all XANES spectra at the energy range -20 to 50 eV, relative to the edge energy, and for all sediment k^3-weighted EXAFS over the k-range 2.5-10.5 Å-1, with the Fe-references identified as the most relevant to fit our experimental data.

References

1. Pennington, S. C. *et al.* EXCHANGE Campaign 1: A Community-Driven Baseline Characterization of Soils, Sediments, and Water Across Coastal Gradients. (2023).
2. Huang, W. & Hall, S. J. Optimized high-throughput methods for quantifying iron biogeochemical dynamics in soil. *Geoderma* **306**, 67–72 (2017).
3. Kropf, A. J. *et al.* The New MRCAT (Sector 10) Bending Magnet Beamline at the Advanced Photon Source. in 299–302 (2010). doi:10.1063/1.3463194.
4. Latta, D. E., Boyanov, M. I., Kemner, K. M., O'Loughlin, E. J. & Scherer, M. M. Abiotic reduction of uranium by Fe(II) in soil. *Appl. Geochem.* **27**, 1512–1524 (2012).
5. Dong, Y. *et al.* Orenia metallireducens sp. nov. Strain Z6, a Novel Metal-Reducing Member of the Phylum Firmicutes from the Deep Subsurface. *Appl. Environ. Microbiol.* **82**, 6440–6453 (2016).
6. Kwon, M. J. *et al.* Impact of Organic Carbon Electron Donors on Microbial Community Development under Iron- and Sulfate-Reducing Conditions. *PLOS ONE* **11**, e0146689 (2016).
7. Flynn, T. M. *et al.* Biogeochemical dynamics and microbial community development under sulfate- and iron-reducing conditions based on electron shuttle amendment. *PLOS ONE* **16**, e0251883 (2021).
8. Ilgen, A. G., Foster, A. L. & Trainor, T. P. Role of structural Fe in nontronite NAu-1 and dissolved Fe(II) in redox transformations of arsenic and antimony. *Geochim. Cosmochim. Acta* **94**, 128–145 (2012).
9. Ravel, B. & Newville, M. *ATHENA*, *ARTEMIS*, *HEPHAESTUS* : data analysis for X-ray absorption spectroscopy using *IFEFFIT*. *J. Synchrotron Radiat.* **12**, 537–541 (2005).
10. Webb, S. M. SIXPack a Graphical User Interface for XAS Analysis Using IFEFFIT. *Phys. Scr.* 1011 (2005) doi:10.1238/Physica.Topical.115a01011.
11. Wilson, M. B., Zhang, C. & Gandhi, J. Analysis of inorganic nitrogen and related anions in high salinity water using ion chromatography with tandem UV and conductivity detectors. *J. Chromatogr. Sci.* **49**, 596–602 (2011).

Supplementary Figures

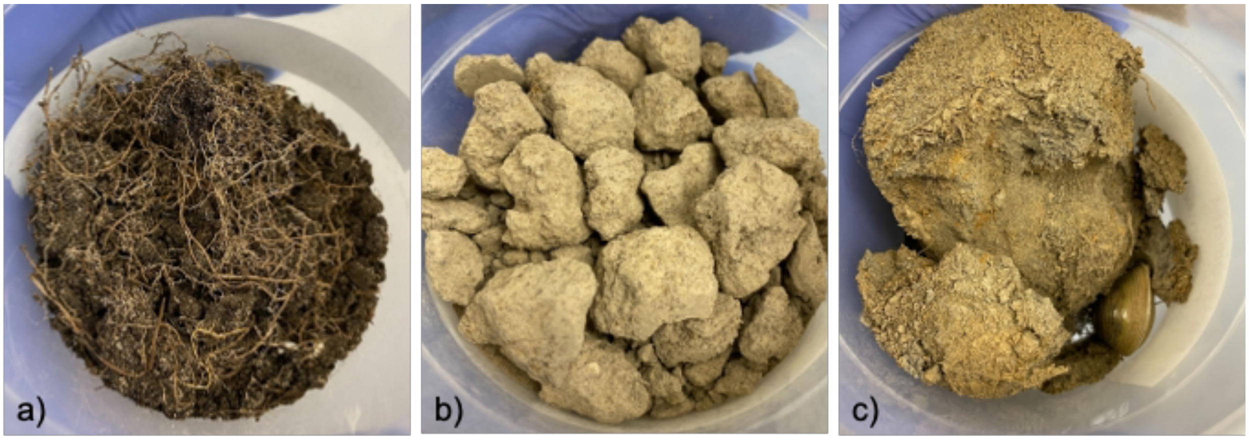

Figure S1. Pictures of lyophilized soils before the dry sieving process. Example of sample characterization from left to right: a) soil sample with moderate root mass/fine roots, b) aggregated and hard to break apart, c) visual iron oxidation and intact shell.

Supplementary Tables

Table S1. List of physical soil characteristics used to index soil and sediment samples.

Physical Soil Characteristics

Soil/Sediment Color	Pebble Shape	Visible Minerals	Presence of Visible Iron Oxidation	Presence of Plastics	Relative Cohesion	Visual Estimation of Sand Content
Light Gray	Rounded	Mica	Yes	Yes	Hard to break apart	100%
Gray	Angular	Quartz	No	No	Easy to break apart	75%
Gray Brown	–	–	–	–	Unconsolidated sand	50%
Light Brown	–	–	–	–	Unconsolidated organic matter	25%
Dark Brown	–	–	–	–	Aggregated	10%
Black	–	–	–	–	–	0%
Reddish	–	–	–	–	–	–
